# Synergistic antibiofilm activity of methylene blue and silver nanoparticle-mediated photothermal therapy against *Enterococcus faecalis* biofilm

**DOI:** 10.1038/s41598-025-29641-8

**Published:** 2025-12-12

**Authors:** Eman M. Fouad, Hossam Tawfiq, Soha Abdelrahman Elhady, Ali M. Saafan

**Affiliations:** 1 Department of Endodontics, Collage of Oral and Dental Surgery, Misr University for Science and Technology (MUST), P.O.Box 77, Giza, Egypt; 2 Faculty of Oral and Dental Medicine, Misr International university, Cairo, Egypt; 3 Medical Microbiology and Immunology, Faculty of Medicine, Ain Shams University, Cairo, Egypt; 4 Dental Laser Applications, NILES, Cairo University, Giza, Egypt

**Keywords:** *E. faecalis*, Biofilm, Photothermal, Silver nanoparticles, Methylene blue, Root canals, Antibiofilm efficiency, Diode laser, Biofilms, Root canal treatment, Dental lasers

## Abstract

Biofilm formation by *Enterococcus faecalis* (*E. faecalis)* in root canals is a significant challenge in endodontic therapy, often leading to persistent infections and treatment failures. This research paper investigates the antibiofilm efficacy of methylene blue mediated photothermal treatment (MB-PTT), as compared to the sole effect of diode laser, PTT, and sodium hypochlorite (NaOCl) on *E. faecalis* biofilms. 45 maxillary central incisors were decoronated, prepared and infected by *E faecalis* for seven days. Forty samples were randomly allocated as follows; GI; irrigated with 2.6% NaOCl, GII; irradiated with 660 nm diode laser (250 mW) for 180 s. GIII; Silver nanoparticles (AgNPs) with diode laser application at same parameters (AgNPs-PTT), GIV: accompanied MB and AgNPs-PTT, while 5 samples were kept as control for biofilm formation. The antibiofilm effect was demonstrated both by bacterial colonies counting (CFU/ml) and scanning electron microscope images. The results highlight the potential of all experimental treatment modalities (*P* < 0.01), However complete absence of detactable bacterial colonies was only evident when MB was coupled with AgNPs-PTT. Accompanied MB with PTT is a promising approach with effective antibiofilm activity against *E. faecalis* biofilms.

## Introduction

The strong association between microorganisms and periapical diseases underscores the central aim of endodontic therapy, which is to eliminate or significantly reduce microorganisms through chemo mechanical root canal debridement. However, achieving effective disinfection is inherently challenging due to the complex anatomy of the pulp space^1^. This challenge is further amplified in regenerative endodontics, where the critical balance between exerting an adequate antibacterial effect and preserving stem cell viability remains a significant obstacle to successful treatment outcomes^2^. This concern has become increasingly relevant given the growing interest in single-visit protocols^3,4^.

Conventional endodontic treatments often rely on potent irrigants such as sodium hypochlorite. While effective, this chemical agent can adversely affect vital tissues and alter the structure of root canal dentin^5^. Moreover, the accumulated body of evidence demonstrated its failure to fully eradicate biofilm^6^. To overcome these limitations, laser technology has emerged as a valuable adjunct for root canal disinfection, offering promising potential in regenerative endeavors^1^. It is believed to possess the capability to access regions that conventional methods might not reach. Laser light is believed to penetrate areas inaccessible to conventional methods^7^, and dentinal tubules have been shown to act as conduits for light transmission^8^.

Silver has long been recognized for its antimicrobial properties, and silver nanoparticles (AgNPs) have further enhanced this effect due to their high surface-area-to-volume ratio^9^. AgNPs exert their antibacterial activity by disrupting bacterial cell walls, generating reactive oxygen species (ROS), and interfering with essential cellular functions, ultimately causing membrane damage, increased permeability, and cell lysis^10^.

Photothermal therapy (PTT), which involves activating metallic nanoparticles with laser energy, was initially developed for cancer treatment^11^. In dentistry, this approach capitalizes on the synergistic antibacterial effects of both laser irradiation and silver nanoparticles through localized heat generation^12^, reaching deeper layers of biofilm^13^.

The integration of metallic nanoparticles, particularly AgNPs, into disinfection protocols has garnered significant scientific interest as researchers seek more effective and biocompatible alternatives^14^. A recent study by Feng et al. demonstrated that Au@Ag core–shell nanoparticles outperformed conventional irrigants, such as sodium hypochlorite and 2% chlorhexidine, while maintaining low cytotoxic profile^10^.

Methylene blue (MB) is an aromatic heterocyclic cationic dye (molecular weight 319.85 g/mol; formula C1_6_H_18_N_3_ClS) with a characteristic absorption peak at 663nm^15^. This classic photosensitizer has been investigated for its ability to enhance the antibiofilm efficacy of light-activated AgNPs^16^. However, the current evidence remains insufficient to fully elucidate its antibiofilm efficiency and the associated topographical changes induced by its effects. The null hypothesis posited that AgNPs-PTT, with or without MB incorporation, would have no additional effect on biofilm eradication compared to laser disinfection alone or standard sodium hypochlorite irrigation.

## Materials and methods

### Sample preparation

This in vitro study was approved by the Research Ethics Committee of the Faculty of Dentistry, Ain Shams University (FDASU-REC), and was registered under the number FDASU-REC-PC012458. The study was conducted in accordance with the CRIS guidelines^17^. Forty-five human maxillary central incisors free from caries, erosion, or external cracks were selected for the study. Teeth exhibiting canal resorption or calcification were excluded. All teeth were extracted for periodontal reasons from patients attending the outpatient clinics of the Faculty of Dentistry, Ain Shams University, after obtaining informed consent. All teeth were immersed in 5.25% NaOCl, washed, and stored in saline. The teeth were then decoronated, giving a fixed root portion length of 16 mm, and root canals enlarged until reaching master apical file #80 (Mani, Japan). External surfaces were sealed with nail varnish and the apex was sealed with composite. Prior to bacterial inoculation, teeth were wrapped in saline-soaked gauze, sterilized by autoclaving for 15 min at 121 °C.

### Bacterial strain and biofilm formation

A clinical strain of *E. faecalis* (ATCC 29212) obtained from the Microbiology laboratory (Central laboratories, Ministry of Health, Egypt) was employed to establish biofilms. The bacterial strain was introduced into Brain Heart Infusion broth (BHI; Difco Laboratories, Dertoit, MI, USA) and cultivated at 37 °C for 24 h. For experimental suspensions, the bacterial marker was cultivated on the surface of Brain Heart Infusion agar under the same incubation conditions. The bacterial cells were then resuspended in saline to achieve an approximate concentration of 3 × 10^8^ cells/ml, adjusted to the No. 1 MacFarland turbidity standard for subsequent sample infection. The teeth were filled with a 24-hour pure culture suspension of *E. faecalis* grown in Brain Heart Infusion broth. All teeth were incubated at 37 °C in sealed vials for 7 days and replenished every 72 h.

### Samples grouping and classifications

The samples were randomly allocated (via closed envelopes) and equally divided into 4 groups as following: G I: 2.6%NaOCl, G II: diode laser, G III: AgNPs-PTT, G IV: MB + AgNPs-PTT. Furthermore, 5 samples were kept aside as positive control and to confirm the formation of biofilm through scanning electron microscope.

### Treatment modality

Group I (NaOCl): Samples were irrigated with 5 ml of 2.6% NaOCl with side vented plastic syringe, 2 mm short of the working length for five minutes. Canals were then flushed with saline.

Group II (Diode laser): Samples were irradiated with diode laser (660 nm) with power output of 250 mW (DPSSL-DRIVER 2, China) at 60 mm distance. The tooth was held in an upright position inside an acrylic block such that the laser beam would penetrate the root canal as deep as possible. The laser machine was activated at the present power for 180 s (continuous wave mode) yielding a total energy of 45 J.

Group III (AgNPs-PTT): Samples were filled with 100 ppm Ag NPs of average size of 15 nm (Nanotech for photo-electronic, Dreamland, Egypt) with a side vented plastic syringe introduced 2 mm short of the working length and left undisturbed for 5 min and then diode laser was applied for 180 s in the same way as mentioned in group 2.

Group IV (MB+AgNPs-PTT): Samples were filled with both 100 µg/ml MB dye (MB, Sigma, USA) and Ag NPs; 0.5 ml of AgNps was introduced first and then the rest of the root canal was filled with MB dye. The diode laser was applied in the same way as mentioned previously.

### Bacterial sampling and counting

Bacterial samples were taken before and after treatment according to the proposed protocol. A sterile paper point was used to absorb and then transfer to a test tube containing 1.0 ml of saline. Each sample was carefully homogenized by being vortexed for 30 s. Serial 10-fold dilution (1:10, 1:100 and 1:1000) was made in saline. Then 0.1 mm from each dilution was smeared to be inoculated on surface of the plate media (BHI agar plates), incubated at 37 °C for 48 h, and colony-forming units (CFU) per 1 ml were enumerated where the number of colonies/plates was multiplied by the corresponding dilution factor and by 10 to determine the total colony forming units (CFU) per ml of sample.

Bacterial reduction was calculated according to following equation^1,18^:

$$Bacterial~reduction\% = \frac{{bacterial~count\left( {pretreatment} \right) - bacterial~count(posttreatment}}{{bacterial~count~(pretreatment}} \times 100$$

On the other hand, Log reduction of bacterial reduction was calculated according to the following formula^19^:.

$$Log{\text{ }}\text{Re} duction{\text{ }} = {\text{ }}\log 10(Bacterial{\text{ }}counts{\text{ }}before{\text{ }}treatment{\text{ }}/{\text{ }}Bacterial{\text{ }}counts{\text{ }}after{\text{ }}treatment)$$

### Scanning electron microscope (SEM)

Samples were scanned for confirmation of biofilm formation and for monitoring morphological changes after each treatment modality. First, samples were longitudinally split and sections with most visible part of apex were chemically prepared by fixing in 3.7% glutaraldehyde in phosphate buffer solution at 4 Cº for 24 h. This was followed by dehydration in graded concentrations of ethanol for 10 min. Finally, samples were air dried, mounted on copper stubs with double sided adhesive tape and coated by gold using Sputter and then examined under the electron microscope (JEOL, JSM-6510LA, analytical scanning electron microscope-Japan) at a standard magnification of 2500X.

### Statistical analysis

Collected data were tabulated and processed using the Statistical Package for Social Science (SPSS) version 26. The effect of the treatment modality is anticipated through comparing bacterial counts samples before and after treatment by paired T-test. The One-Way ANOVA test was employed for assessing significant differences among means of bacterial reduction of study groups while Post Hoc Tests were utilized when differences among means were identified. Pairwise multiple comparisons tested the difference between each pair of means, and yielded a matrix where asterisks indicate significantly different group means at an alpha value of 0.05.

## Results

### Antibiofilm efficacy

All study groups exhibited a significant reduction in biofilm viability (*p* < 0.01) as shown in Table 1. The highest mean value of bacterial reduction was revealed in GIV while the lowest was GI (Table 2). The bacterial CFU counts of GI was statistically the highest among all study groups. The AgNPs mediated PTT group (GIII) demonstrated a notable decrease in biofilm viability that was statistically comparable to the laser group (GII), however, this effect was further enhanced in the MB + AgNPs-PTT group (GIV) where no detectable CFU was observed. 

**Table 1:** Bacterial counts (CFU/ml) means and standard deviation (SD) of study groups.

Study group	Subgroup	Mean (X^3^) ± SD	*P* value
GI (NaOCl)	Pretreatment	136.7 ± 28.3	< 0.01*
	Posttreatment	0.27 ± 0.1	
GII (Diode Laser)	Pretreatment	131.1 ± 29.8	< 0.01*
	Posttreatment	0.156 ± 0.052	
GIII (AgNPs-PTT)	Pretreatment	135.6 ± 29.6	< 0.01*
	Posttreatment	0.12 ± 0.07	
GIV (MB + AgNPs-PTT)	Pretreatment	138.9 ± 28.5	< 0.01*
	Posttreatment	00.0 ± 0	

*Statistical significance at P value ≤ 0.05.

**Table 2:** Bacterial reduction and log reduction of *E. faecalis* means (X^3^) and standard deviation (SD) of study groups.

Study group	Mean ± SD	*P* value	Mean (%) ± SD	Log reduction
GI (NaOCl)	0.27 ± 0.1 ^a^	< 0.01*	99.7 ± 0.001	2.7
GII (Diode Laser)	0.156 ± 0.05 ^b^		99.8 ± 0.001	2.9
GIII (AgNPs-PTT )	0.12 ± 0.07 ^b^		99.9 ± 0.001	3.05
GIV (MB + AgNPs-PTT)	00.0 ± 0 ^c^		100 ± 0.00	∞

*Statistical significance at P value ≤ 0.05.

Different letters reveal statistical difference.

### Scanning electron microscope (SEM) analysis

**Fig. 1:**
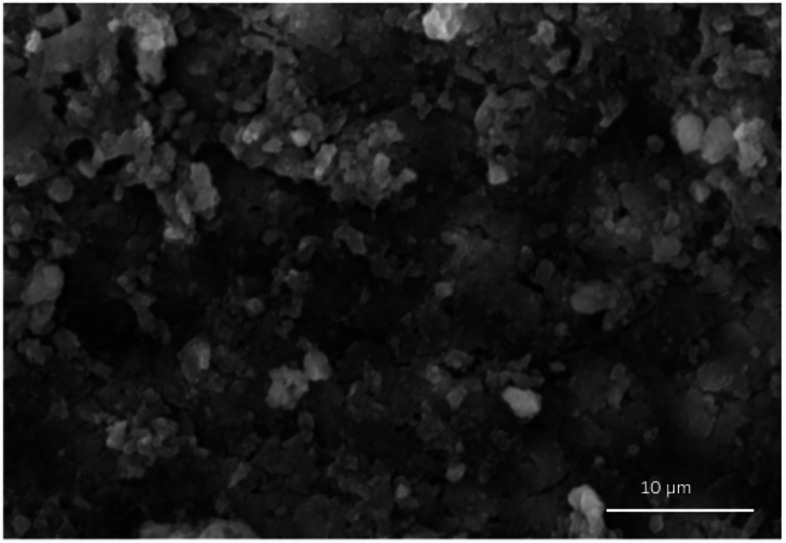
SEM micrograph at magnification of 2500X confirming the formation of the biofilm, where the bacterial colonies extend along the surface of the dentine.

**Fig. 2:**
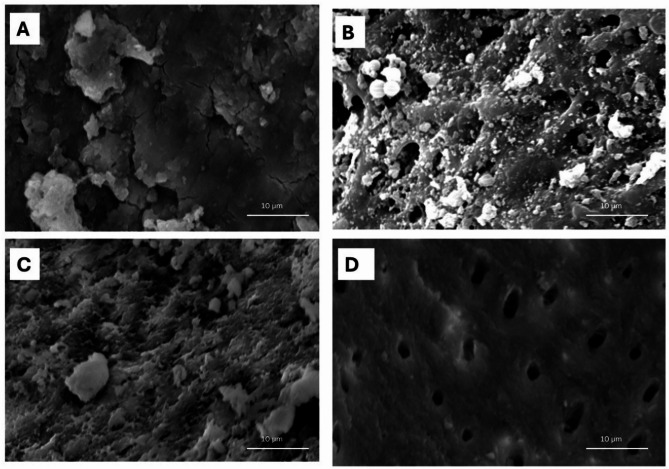
SEM micrograph at magnification of 2500X showing the topographic changes on the dentin surface and the effect on opening of dentinal tubules of the treatment groups; GI, GII, GIII, GIV respectively (from top left to bottom right).

The SEM micrographs confirmed the formation of biofilms where E. faecalis colonies colonized the entire dentinal surface as shown in Fig. 1. A dense and heterogeneous smear layer was covering the entire dentine surface and occluding dentinal tubules in samples irrigated of GI (Fig. 2A). Disruption of biofilm architecture and thickness in both G II and GIII (Fig. 2B and 2C respectively) was evident. These groups demonstrated partial smear layer removal, though residual debris remained, indicating moderate effect without complete dentinal tubule exposure. The most pronounced antibiofilm effect was observed where PTT was accompanied by MB as dentine surface was clear, showing dentinal tubules opening, and smear layer was totally deficient (Fig. 2D).

## Discussion

This study investigated the antibacterial efficacy and surface topography alterations of dentine following methylene blue (MB)-mediated photothermal therapy (PTT) enhanced with metallic nanoparticles. The synergistic effects of this combination were evaluated in comparison to diode laser irradiation alone, nanoparticle-mediated PTT, and conventional sodium hypochlorite irrigation.

*Enterococcus faecalis*, a facultative Gram-positive bacterium commonly associated with persistent endodontic infections, was chosen due to its known resistance to antimicrobial agents and its capacity to form biofilms capable of penetrating dentinal tubules to depths exceeding 1000 μm^7^. In fact, the biofilm structure per se is blamed, involving mechanisms such as quorum sensing, metabolic dormancy, and efflux pump activity, which contribute to its resilience^20,21^. Bacterial reduction was assessed using colony-forming unit (CFU) counts, a reliable and cost-effective method^22^. After one week of incubation, biofilm formation was confirmed on the root surfaces, and the observed scanning electron micrographs closely resembled those reported in the literature^23^.

Root canal instrumentation was standardized to a relatively large apical size to ensure uniform laser energy distribution, thereby enhancing the effectiveness of laser-based disinfection modalities^24^. Direct laser irradiation enables more consistent exposure across the canal walls, ensuring a more effective disruption of the bacterial biofilms, with potential inconsistency and power loss of light energy across fiber-optic within different laser settings^25^. Additionally, large canals in this study mirror the conditions of regenerative endodontics, where MB mediated laser disinfection has shown promise to support tissue regeneration aided with adequate disinfection of the wide canal space inherent to immature teeth^2^. EDTA was not employed in this study to avoid introducing a confounding variable that could bias the topographical changes by smear layer removal. Leaving the smear layer intact presented a more stringent and clinically relevant challenge, due to occlusion of dentinal tubules hindering the penetration of disinfecting agents^2,26^.

The application of nanotechnology in endodontics is a rapidly evolving field, evidenced by a high density of citations, with *E. faecalis* biofilms forming the core of bibliometric analyses since 2000^27^. Silver nanoparticles have emerged as a promising antimicrobial agent, significantly enhancing bacterial eradication due to their high surface reactivity and metallic properties^28^. Notably, AgNPs amplify singlet oxygen production, yielding a fourfold increase in treatment efficiency^29^. Additionally, their demonstrated hemocompatibility and reduced cytotoxicity make them suitable for biologically based, single-visit endodontic procedures^30^.

Our results aligned with those of Hendi et al., who reported that laser-irradiated AgNPs achieved greater disinfection than either treatment alone^31^. Discrepancies in NaOCl efficacy across studies may be attributed to variations in concentration (5% vs. 2.6%) and irradiation protocols, such as shorter exposure durations (three successive cycles of 15 s each) in the previous study. Comparative results were manifested with Erbium Chromium Laser^32^. Our results also agree with those of Obeid and Nour regarding diode laser effectiveness^33^, but contrasts with Sohrabi et al., who found high-concentration NaOCl to be superior^34^.

Methylene blue is a hydrophilic photosensitizer with low dark toxicity^35^, was used at 100 µg/mL to balance antimicrobial efficacy and biocompatibility while avoiding the shielding effects seen at higher concentrations^2,36^. The thick peptidoglycan and lipoteichoic acid layers of E. faecalis facilitate the diffusion of MB and nanoparticles, enhancing the synergistic antimicrobial effect^37^.

Notably, the most profound antibacterial and topographical changes were observed in Group IV, treated with MB-mediated PTT. SEM analysis revealed complete smear layer removal and open dentinal tubules, indicating deep biofilm reduction. These findings are consistent with previous studies showing enhanced antibiofilm activity of light-activated nanosilver hydrogels and gold nanorods^10,38,39^. Additionally, emerging evidence suggests that the combination of PTT and MB enhances efficacy against cancer cells, which may have implications for biofilm-targeted therapies^40^. The metallic properties of nanosized particles could potentially amplify the delivery of photoenergy and the nanosized particles enrollment increased efficiency of PTT and internalization of the targeted microorganisms^28^. The enhanced antibacterial activity can be explained by their complementary mechanisms of action. Silver nanoparticles themselves can act as photosensitizers, producing reactive oxygen species when activated by laser light^41^. This effect is markedly amplified in the presence of MB, a cationic photosensitizer with strong affinity for the negatively charged cell walls of E. faecalis^42^. Upon irradiation, MB undergoes photodynamic activation through two major pathways: type I reactions, involving electron transfer and free radical formation, and type II reactions, involving energy transfer and the production of singlet oxygen^43^. Both pathways generate highly reactive species that induce oxidative stress, lipid peroxidation, and hydroperoxide formation, ultimately compromising bacterial membranes and leading to cell death^41^. Importantly, Gram-positive bacteria such as E. faecalis are particularly susceptible to this mechanism due to their thick peptidoglycan and teichoic acid–rich cell walls, which facilitate MB binding and penetration^37,44^. Beyond the classical photodynamic mechanism, silver nanoparticles contribute an additional dimension through their plasmonic properties. When exposed to red laser light, surface electrons of AgNPs oscillate collectively in a phenomenon known as surface plasmon resonance (SPR). This greatly enhances light absorption and generates localized electromagnetic fields, allowing AgNPs to function as efficient “light harvesters”^45^. The plasmonic response not only promotes more efficient excitation of MB but also catalyzes partial photodegradation of MB molecules, leading to sustained oxygen activation and hydroxyl radical production^15^. In parallel, the absorbed energy is converted into localized heat, exerting a photothermal effect that disrupts bacterial biofilm architecture^46^. Collectively, these synergistic mechanisms account for the superior antibacterial and antibiofilm efficacy observed in the MB–AgNPs-PTT group. 

Our observations also support findings by Afkhami et al. on the efficacy of nanoparticle-assisted photodynamic therapy, though methodological differences—including photosensitizer type namely indocyanine green (ICG), irradiation parameters (200 mW for 30 s), and light delivery system—likely account for variations in outcomes. This highlights the efficiency of direct laser irradiation over the end-firing fiber-optic delivery in certain settings^16^. The synergistic effects observed here also correspond with recent studies on photo-sonodynamic therapy using MB-incorporated nanoparticles, which outperformed laser irradiation, ultrasonics, or MB-Photodynamic therapy alone^37^. Therefore, the null hypothesis was rejected, as the MB-PTT combination demonstrated significantly superior antibacterial effect.

The integration of MB with PTT and direct laser irradiation represents a promising strategy for improving disinfection outcomes, especially in regenerative endodontics involving immature teeth with large canals. However, several limitations must be acknowledged. Within the limitation of CFU-based detection which can not account for viable but not culturable cells (VBNC), the present findings should be interpreted cautiously. The in vitro model does not fully replicate the complexity of polymicrobial clinical infections. Additionally, the direct laser application used in this study may not be feasible in clinical scenarios involving coronal structures and curved canals. Finally, the long-term clinical safety and efficacy of silver nanoparticles warrant further investigation through well-designed clinical trials.

## Conclusion

Silver nanoparticles mediated photothermal therapy has a significant antibiofilm efficiency against *E. faecalis* biofilms in root canals. Further enhancement is achieved through incorporation of MB leading to marked biofilm reduction . This suggests the potential application of the innovative approach for biofilm destruction improving endodontic treatment outcomes. Within the limitations of CFU-based detection, future studies incorporating advanced methods, alongside in vivo validation, are warranted to better confirm its translational potential.

## Data Availability

Data are available from the corresponding author upon reasonable request.

## Abbreviations

*E. faecalis*: *Enterococcus faecalis*
MB: Methylene blue
PTT: Photothermal therapy
AgNPs: Silver nanoparticles
SEM: Scanning electron microscope
CFU: Colony forming units
